# Comparison of Lobectomy and Sublobar Resection for Stage IA Elderly NSCLC Patients (≥70 Years): A Population-Based Propensity Score Matching’s Study

**DOI:** 10.3389/fonc.2021.610638

**Published:** 2021-05-07

**Authors:** Bo Zhang, Renwang Liu, Dian Ren, Xiongfei Li, Yanye Wang, Huandong Huo, Shuai Zhu, Jun Chen, Zuoqing Song, Song Xu

**Affiliations:** ^1^ Department of Lung Cancer Surgery, Lung Cancer Institute, Tianjin Medical University General Hospital, Tianjin, China; ^2^ Tianjin Key Laboratory of Lung Cancer Metastasis and Tumour Microenvironment, Lung Cancer Institute, Tianjin Medical University General Hospital, Tianjin, China

**Keywords:** NSCLC, sub-lobar resection, lobectomy, propensity score matching, SEER

## Abstract

**Background:**

To investigate the differences in survival between lobectomy and sub-lobar resection for elderly stage I non-small-cell lung cancer (NSCLC) patients using the Surveillance, Epidemiology, and End Results (SEER) registry.

**Method:**

The data of stage IA elderly NSCLC patients (≥ 70 years) with tumors less than or equal to 3 cm in diameter were extracted. Propensity-matched analysis was used. Lung cancer-specific survival (LCSS) was compared among the patients after lobectomy and sub-lobar resection. The proportional hazards model was applied to identify multiple prognostic factors.

**Results:**

A total of 3,504 patients met criteria after propensity score matching (PSM). Although the LCSS was better for lobectomy than for sub-lobar resection in patients with tumors ≤ 3 cm before PSM (p < 0.001), no significant difference in the LCSS was identified between the two treatment groups after PSM (p = 0.191). Multivariate Cox regression showed the elder age, male gender, squamous cell carcinoma (SQC) histology type, poor/undifferentiated grade and a large tumor size were associated with poor LCSS. The subgroup analysis of tumor sizes, histologic types and lymph nodes (LNs) dissection, there were also no significant difference for LCSS between lobectomy and sub-lobar resection. The sub-lobar resection was further divided into segmentectomy or wedge resection, and it demonstrated that no significant differences in LCSS were identified among the treatment subgroups either. Multivariate Cox regression analysis showed that the elder age, poor/undifferentiated grade and a large tumor size were a statistically significant independent factor associated with survival.

**Conclusion:**

In terms of LCSS, lobectomy has no significant advantage over sub-lobar resection in elderly patients with stage IA NSCLC if lymph node assessment is performed adequately. The present data may contribute to develop a more suitable surgical treatment strategy for the stage IA elderly NSCLC patients.

## Introduction

Lung cancer is a major public health problem worldwide and is the leading cause of death in the United States (1). With the extensive use of low-dose CT, the early detection rate of lung cancer has increased, and the mortality rate of lung cancer has decreased remarkably (2). Approximately 228 000 people in the United States were diagnosed with lung cancer in 2019, and lung cancer accounts for 40% of cancer-related deaths (1). Among the common subtypes of lung cancer, non-small-cell lung cancer (NSCLC) represents 85% of lung cancer cases (3). Approximately three-quarters of lung cancer survivors are aged 65 years or older (4). Previous numerous studies defined elderly patients as those age 70 years or older (5, 6), so we mainly focus on this population. Surgical resection plays an extremely important role in the early stage of lung cancer. Lobectomy shows better survival than sub-lobar resection for patients with NSCLC tumors ≤ 1 cm and > 1 to 2 cm (7). However, controversy still remains about the extent of appropriate resection for early stage lung cancer, and there are many salient arguments both for and against lobectomy and sub-lobar resection. Sub-lobar resection procedures, including wedge resection and segmentectomy, have been reported as an alternative surgical technique. There is an increasing amount of evidence that sub-lobar resection, when applied in appropriate patient populations, can provide not only excellent oncologic results but also no significant difference survival to lobectomy. Limited resection is adequate for the management of small-sized adenocarcinomas (≤ 2 cm) of the lung (8). A *post hoc* analysis of an international, randomized, phase 3 trial showed that the morbidity rates did not seem to differ between lobectomy and sub-lobar resection for early-stage (≤ 1 cm) NSCLC (9). A retrospective study indicated that sub-lobar resection might achieve similar survival rates to lobectomy in elderly stage I NSCLC patients (10). A result was observed in that segmentectomy failed to show superiority in terms of survival compared with wedge resection for patients with stage I NSCLC (11). Thus, the optimal extent of resection for elderly patients with stage IA disease remains unclear. Compared with young lung cancer patients, elderly patients more often have underlying disease and poor pulmonary function. Multiple preoperative comorbidities or poor lung function lead to high morbidity and mortality rates, which severely limits optimal treatment planning (12). Sub-lobar resection, including segmentectomy and wedge resection, has the advantages of better preserving postoperative lung function, fewer complications, and a lower mortality rate (13). Lung cancer is a chronic disease in the elderly population. The incidence of lung cancer gradually increases with age. Sub-lobar resection has emerged as a replacement for lobectomy for the treatment of early-stage NSCLC.

In this study, we aimed to investigate the outcomes of sub-lobar resection versus lobectomy in elderly patients with stage IA NSCLC using the population-based Surveillance, Epidemiology, and End Results (SEER) registry, and we attempted to propose the optimal surgical management for this population.

## Materials and Methods

### Patients

We selected patients from the SEER database. Patients were included in our study if the following inclusion criteria were met: (1) pathologically stage of TNM staging AJCC sixth or seventh edition and then adjusted manually according to the AJCC eighth edition criteria, namely stage IA NSCLC with tumors ≤ 3 cm between January 2004 and December 2015; (2) aged ≥ 70 years; (3) active follow-up after surgery; and (4) presence of one malignant primary lesion. To restrict the data set for NSCLC, we excluded patients with small cell carcinoma and tumors located bilaterally and in the main bronchus. Patients were also excluded if they received chemotherapy or radiotherapy preoperatively, intraoperatively, or postoperatively or if their operation information was unknown. The patients with invading the visceral pleura, atelectasis or obstructive pneumonia were also not included.

The demographics of the patients (age, marital status, gender, and race/ethnicity), characteristics of the tumors (size, location, grade, and histologic type), cause-specific death classification, dissected regional lymph nodes and treatment details (surgical type) were collected from the SEER database. In this study, the histologic subtypes were classified as squamous cell carcinoma, adenocarcinoma and other histologic types (e.g., large-cell carcinoma, papillary cell carcinoma). Patients were divided into lobectomy and sub-lobar resection (wedge and segmentectomy) groups according to the surgical procedure. We used propensity score matching (PSM) to minimize the effect of potential confounders that existed in the baseline characteristics of patients in different treatment groups. Our primary outcome of interest was lung cancer–specific survival (LCSS) after PSM according to specific codes provided by SEER. LCSS was defines as the date of surgery to the date of lung cancer–specific death.

### Statistical Analysis

We used the t test and chi-square test to compare differences between continuous and categorical variables. Kaplan-Meier (KM) survival analysis using the log rank test was used to assess the differences in LCSS. The surgical allocation of the SEER database was not randomly assigned for the study population; therefore, we used propensity score matching (PSM) to balance the pretreatment variables to lower the selection bias. Variables included in the PSM model were selected from the available clinicopathological characteristics that were associated with the surgical choice and/or study outcomes: age, gender, race/ethnicity, marital status, tumor location, laterality, tumor size, dissected regional lymph nodes, and tumor histologic type. We created 1:1 matched cohorts by matching patients who underwent lobectomy and sub-lobar resection and used the log-rank test to compare the survival curves between lobectomy and sub-lobar resection by tumor size (T ≤ 1 cm, > 1 cm to 2 cm and > 2 cm to 3 cm), regional LNs resection (no-LN dissection, 1-3 regional LNs or ≥ 4 regional LNs) and histologic type (squamous cell carcinoma, adenocarcinoma and other carcinomas). We performed a subgroup analysis of lymph node status regardless of its univariate significance given the clinical and prognostic importance of lymph node involvement. We created a balanced cohort using an optimized performance-matching algorithm with a caliper setting of 0.01. The balances of matched covariates were measured by the standardized mean difference, and a difference between -0.1 and 0.1 was generally considered negligible (14). A Cox proportional hazards model that included all of the best subsets of predictors from the SEER database was applied to adjust for baseline variables in the comparison. A two-sided P value of < 0.05 was considered statistically significant. All analyses were conducted with SPSS 24.0 (SPSS, Chicago, IL, USA), and the survival curve was made with Stata 15 (StataCorp LP, College Station, TX, USA).

## Results

### Patient Demographics and Survival Analysis

**Figure 1:**
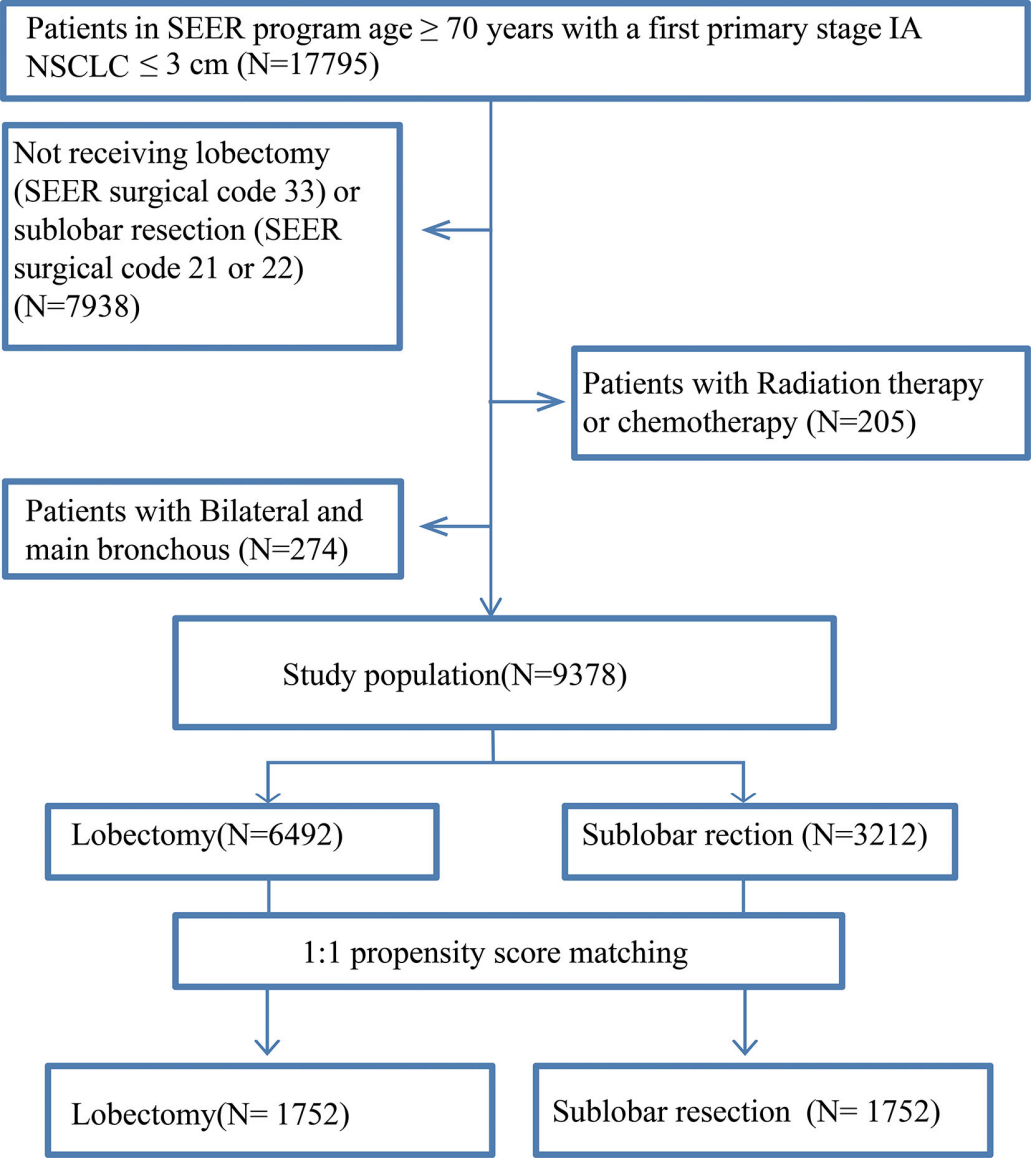
Selection process of eligible patient.

**Figure 2:**
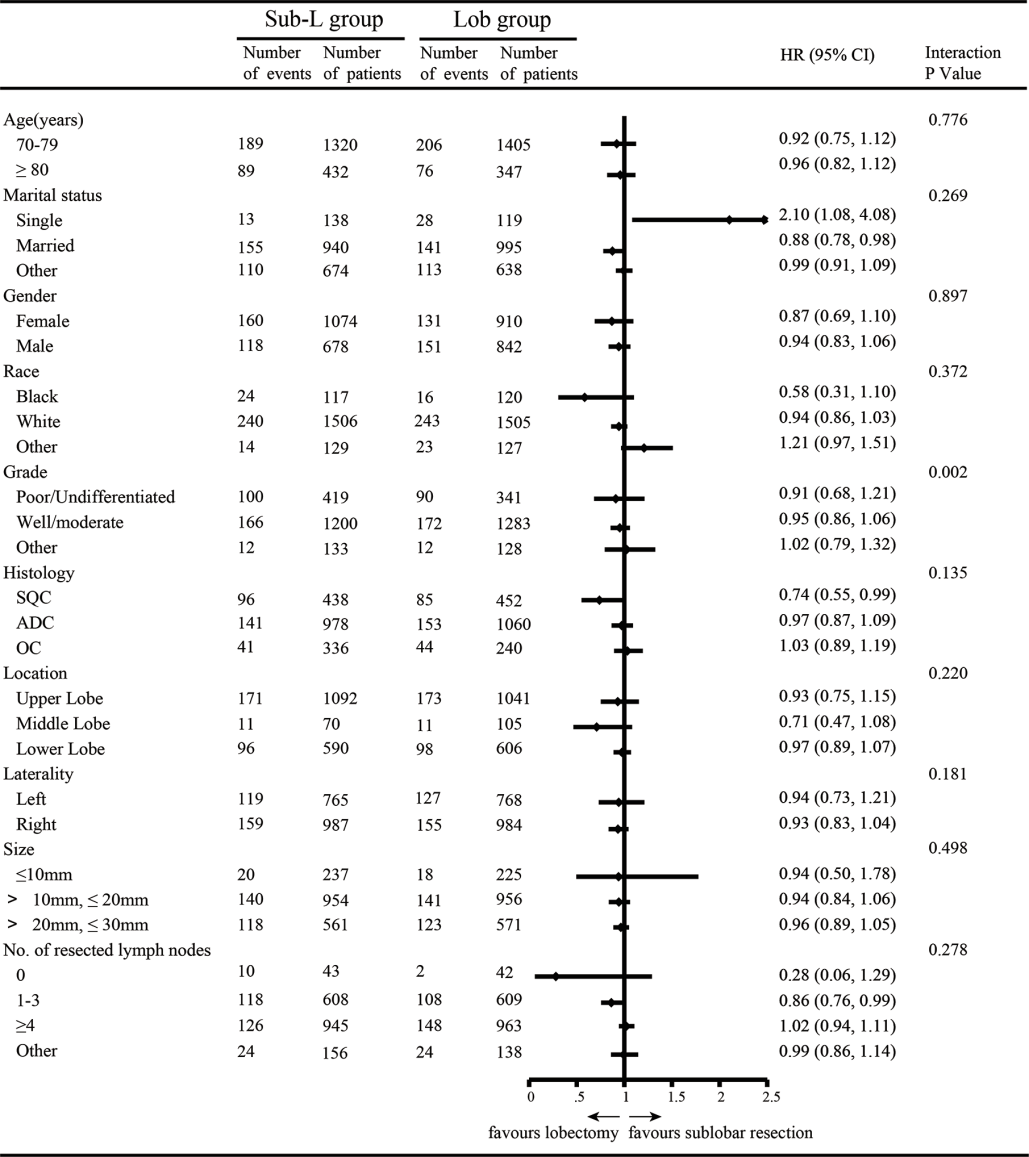
Subgroup analysis by independent review undergoing lobectomy (Lob) and sub-lobar resection (Sub- L).

After PSM, 3,504 elderly T1N0M0 NSCLC patients were enrolled: 1,752 patients underwent lobectomy, and 1,752 patients underwent sub-lobar resection. For the patients who underwent sub-lobar resection, 493 (28.14%) underwent segmentectomy, and 1,259 (71.86%) underwent wedge resection. A complete flow chart of the selection process is shown in **Figure 1**. The median survival time was 91 months in the lobectomy group and 80 months in the sub-lobar resection group. The baseline characteristics are listed in **Table 1**, respectively. In terms of marital status and gender, there was no significant difference before PSM between the groups (**Table 1**). Nevertheless, there were no significant differences in marital status, race, laterality, tumor size, No. of resected lymph nodes after PSM (**Table 1**). The values of standardized mean difference (Smd) showed were between -0.1 to 0.1, which showed that the groups were well-balanced after PSM (**Table 1**). In LCSS analysis, the p-value of the interaction test showed only grade was less than 0.05 (**Figure 2**). 1340 patients who underwent sub-lobar resection were included after PSM, of whom 670 received segmentectomy and 670 received wedge resection. Analysis showed there were no significant difference in age, gender, race and histology before PSM between the groups (**Supplementary Table 1**). Similarly, there was no significant difference in age, marital status, gender, race, laterality, histology and size after PSM between the groups (**Supplementary Table 1**). The values of standardized mean difference were between -0.1 to 0.1 after PSM (**Supplementary Table 1**). The interaction test between segmentectomy and wedge resection was also performed with the p-value of grade, location, laterality and No. of resected lymph nodes for LCSS being less than 0.05 (**Supplementary Figure 1**).

**Table 1:** Baseline characteristics for overall survival in patients with NSCLC ≤ 3 cm.

	No. (%) of Patients before PSM				No. (%) of Patients after PSM			
	Sub-L (N=3212)	Lob (N=6492)	P	Smd	Sub- L (N=1752)	Lob (N=1752)	P	Smd
Age (Median)			<0.001				0.001	
≥70 to 79y	2289 (71.2)	5230 (80.5)		-0.218	1324 (75.5)	1405 (80.1)		-0.098
≥80y	923 ( 28.8)	1262 (19.5)		0.218	428 (24.5)	347 (19.9)		0.098
Marital status			0.052				0.197	
Single	222 (6.9)	462 (7.1)		-0.007	136 (7.7)	119 (6.7)		0.038
Married	1702 (53)	3592 (55.4)		-0.048	945 (53.9)	995 (56.8)		-0.058
Other	1288 (40.1)	2438 (37.5)		0.053	471 (38.2)	638 (36.5)		0.035
Gender			0.421				<0.001	
Female	1912 (59.5)	3809 (58.7)		0.016	1070 (59.5)	910 (54.9)		0.093
Male	1300 (40.5)	2683 (41.3)		-0.016	682 (40.5)	842 (45.1)		-0.093
Race			0.001				0.891	
Black	192 (5.9)	332 (5.2)		0.030	117 (6.6)	120 (6.8)		-0.008
White	2799 (87.2)	5583 (86)		0.035	1501 (85.7)	1505 (85.9)		-0.005
Other	221 (6.9)	577 (8.8)		-0.070	134 (7.7)	127 (7.3)		0.015
Grade			0.007				0.004	
Poor/Undifferentiated	796 (24.8)	1522 (23.4)		0.032	420 (23.7)	134 (19.7)		0.097
Well/moderate	2161 (67.3)	4546 (70.1)		-0.060	1201 (68.8)	1283 (73)		-0.092
Other	255 (7.9)	424 (6.5)		0.054	131 (7.5)	128 (7.3)		0.007
Histology			< 0.001				0.009	
SQC	844 (26.3)	1521 (23.4)		0.067	432 (24.6)	452 (25.7)		-0.025
ADC	1804 (56.2)	4072 (62.8)		-0.134	1014 (58.1)	1060 (60.4)		-0.040
OC	564 (17.5)	899 (13.8)		0.101	306 (17.3)	240 (13.9)		0.093
Location			0.004				0.020	
Upper Lobe	1959 (61)	4013 (61.8)		-0.016	1080 (61.7)	1041 (59.6)		0.042
Middle Lobe	141 (4.3)	375 (5.7)		-0.064	70 (4.1)	105 (5.9)		-0.082
Lower Lobe	1112 (34.7)	2104 (32.5)		0.046	600 (34.2)	606 (34.5)		-0.006
Laterality			<0.001				0.919	
Left	1443 (44.9)	2584 (39.8)		0.103	765 (43.6)	768 (43.8)		-0.004
Right	1769 (55.1)	3908 (60.2)		-0.103	987 (56.4)	984 (56.2)		0.004
Size			<0.001				0.935	
≤10mm	585 (18.2)	491 (7.5)		0.323	228 (13)	225 (12.8)		0.005
>10mm, ≤20mm	1793 (55.9)	3153 (48.5)		0.148	963 (54.9)	956 (54.7)		0.004
>20mm, ≤30mm	834 (25.9)	2848 (44)		-0.386	561 (32.1)	571 (32.5)		-0.008
No. of resected lymph nodes			<0.001				0.686	
0	1333 (41.5)	42 (0.6)		1.159	45 (2.5)	42 (2.4)		0.006
1-3	771 (24)	709 (10.9)		0.350	601 (34.4)	609 (34.8)		-0.008
≥4	951 (29.7)	5512 (85)		-1.348	949 (54.2)	963 (54.9)		-0.014
Other	157 (4.8)	229 (3.5)		0.065	157 (8.9)	138 (7.9)		0.036

Lob , lobectomy ; Sub-L , sub-lobar resection ; SQC , Squamous carcinoma ; ADC , adenocarcinoma ; OC , other carcinoma ; PSM , propensity score matching ; Smd , standardized mean differences.

**Figure 3:**
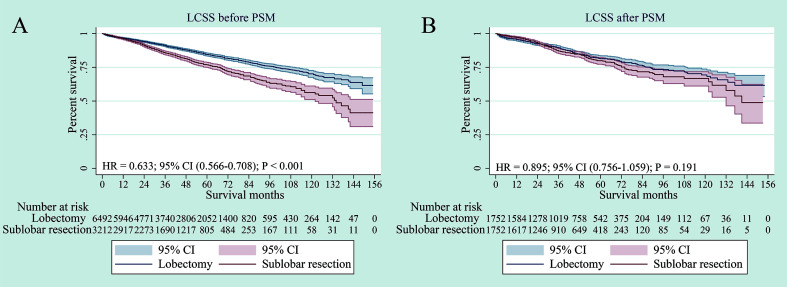
Lung cancer–specific survivals in patients with NSCLC ≤ 3 cm undergoing lobectomy or sub-lobar resection before PSM **(A)** or after PSM **(B)**.

The survival analysis showed that compared to sub-lobar resection, lobectomy had significant advantage over sub-lobar resection for LCSS before PSM (p < 0.001) (**Figure 3A**). However, in terms of LCSS, the two surgical approaches had no substantial difference after PSM (p = 0.191) (**Figure 3B**). When specifically comparing wedge resection *vs* segmentectomy, segmentectomy was also not superior to wedge resection in terms of LCSS after PSM (p = 0.154) (**Supplementary Figure 2B**).

### Subgroup Analysis According to Tumour Size

**Figure 4:**
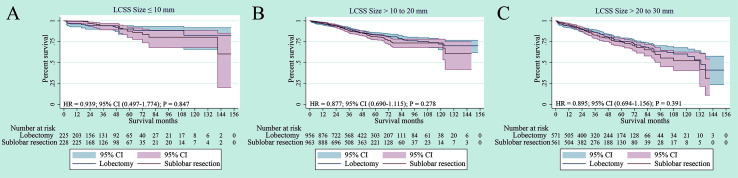
Lung cancer–specific survivals in patients with NSCLC after PSM (NSCLC) ≤1 cm **(A)**, NSCLC > 1 to 2 cm **(B)** or NSCLC > 2 to 3cm **(C)** undergoing lobectomy and sub-lobar resection.

In the NSCLC 8^th^ TNM staging system, T1 tumors were classified into T1mi, T1a (≤ 1 cm), T1b (1-2 cm) and T1c (2-3 cm) tumors (15). We further performed subgroup survival analysis according to tumor size. There was no significant difference in terms of LCSS for NSCLC patients with tumors size ≤ 1 cm, > 1 to 2 cm and > 2 to 3 cm between lobectomy and sub-lobar resection (p = 0.847, p =0.278 and p =0.391, respectively). (**Figures 4A–C**). For tumors ≤ 1 cm, > 1 to 2 cm and > 2 to 3 cm, segmentectomy and wedge resection showed no significant differences in LCSS (p = 0.363, p = 0.091 and p = 0.429, respectively) (**Supplementary Figure 3**). In terms of tumor sizes, these results suggest that lobectomy may not prolong cancer-related survival for elderly patients with stage IA NSCLC.

### Subgroup Analysis According to Histology

**Figure 5:**
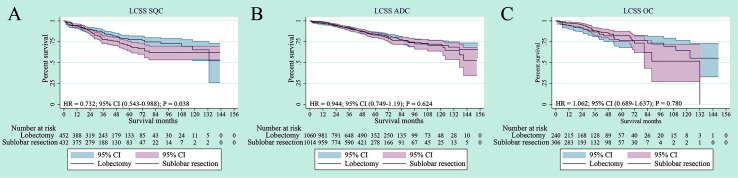
Lung cancer–specific survivals in patients with NSCLC after PSM [Histology type: SQC **(A)**, ADC **(B)** or OC **(C)**] undergoing lobectomy and sub-lobar resection.

The survival analyses were also investigated according to histology, including squamous cell carcinoma, adenocarcinoma and other carcinoma histologic types. Although lobectomy was associated with an LCSS superior to sub-lobar resection in the SQC, no significant statistical difference was found by adjusted subgroup analysis (**Figure 5A**). In terms of ADC and OC, there was also no statistical difference in LCSS (p = 0.624, p = 0.780, respectively) (**Figures 5B, C**).

In addition, we observed that all histology types had no significant difference for LCSS between segmentectomy and wedge resection (p = 0.070, p = 0.364 and p = 0.697, respectively) (**Supplementary Figures 4A–C**).

### Subgroup Analysis According to Regional LN Dissection

**Figure 6:**
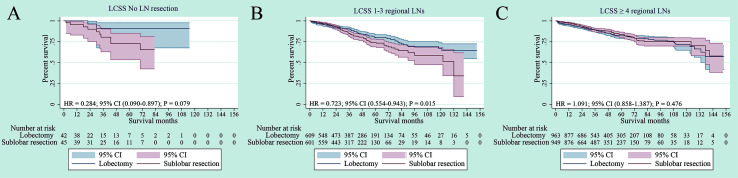
Subgroup analysis of cancer-specific survival after PSM following lobectomy and sublobar resection for lung cancer with tumor size ≤ 3 cm. [Lymph nodes (LN) resection: no LN resection **(A)**, 1-3 LN resection **(B)** or ≥ 4 LN resection **(C)**].

The survival analyses were also investigated according to regional lymph node (LN) dissection. Patients were classified into no-LN dissection, 1-3 regional LNs or ≥ 4 regional LNs. No difference was observed in LCSS for patients with no-LN dissection between sub-lobar resection and lobectomy (p = 079) (**Figure 6A**). For 1-3 regional LNs, lobectomy was associated with an LCSS superior to sub-lobar resection (p = 0.015) (**Figure 6B**). In terms of ≥ 4 regional LNs dissection, no difference in LCSS was identified between sub-lobar resection and lobectomy (p = 0.476) (**Figure 6C**). When specifically comparing wedge resection *vs* segmentectomy, no significant difference was observed in LCSS among no-LN dissection, 1-3 regional LNs and ≥ 4 regional LNs (all p > 0.05) (**Supplementary Figure 4**).

### Cox Regression Analysis

**Figure 7:**
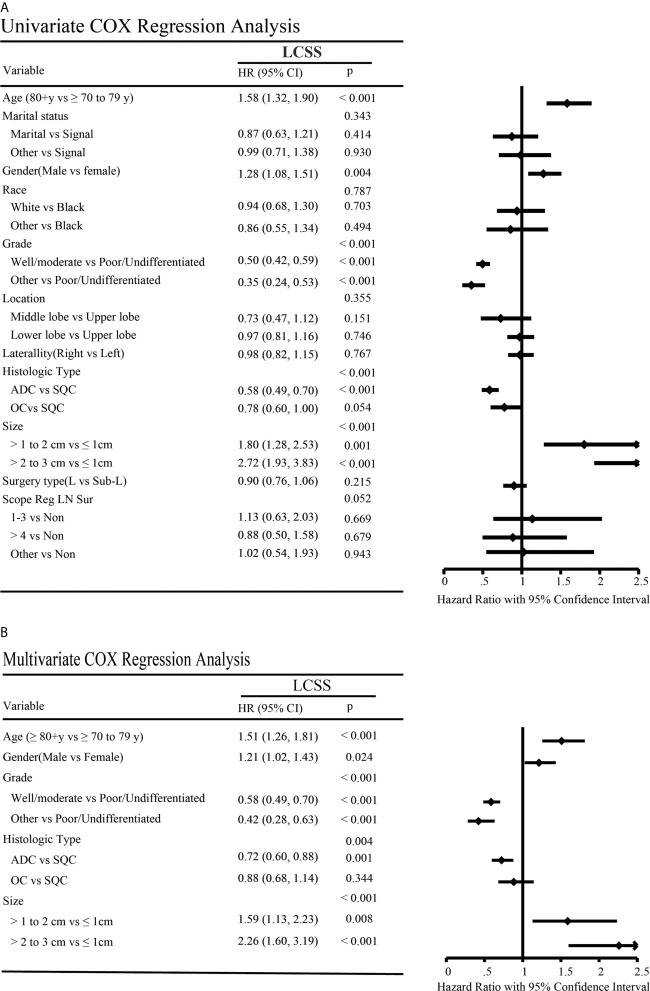
Univariate **(A)** and multivariate **(B)** Cox regression analysis of factors affecting lung cancer-specific survivals.

The Cox proportional hazards regression model was applied to investigate the potential confounding factors related to LCSS between lobectomy and sub-lobar resection for elderly NSCLC patients with a tumor size ≤ 3 cm (**Figure 7**). Univariate analysis revealed that LCSS was significantly higher in the older age, poor/undifferentiated grade, males gender, SQC histology type and in those with larger tumor size (all p < 0.05), but patient marital status, race, location, laterality and surgery type were not significantly correlated with LCSS (**Figure 7A**). Similarly, multivariate Cox regression analysis showed the elder age, male gender, poor/undifferentiated grade, SQC histology type and a large tumor size were associated with poor LCSS (**Figure 7B**).

In addition, surgical subgroups, including segmentectomy and wedge resection, of the Cox proportional hazards regression model were also performed (**Supplementary Figure 6**). Univariate analyses revealed that the elder age, male gender, poor/undifferentiated grade, SQC histology type, large tumor size and No. of regional LNs dissection were significantly associated with patient LCSS (p < 0.05) (**Supplementary Figure 6A**). Multivariate Cox regression analysis showed that the elder age, male gender, poor/undifferentiated grade and large tumor size were a statistically significant independent factor associated with survival (**Supplementary Figure 6B**).

## Discussion

Lobectomy is considered the standard of care for operable early stage NSCLC (16). However, surgeons are often reluctant to recommend lobectomy for patients who are older, who have comorbid conditions or poor pulmonary function. Therefore, there is still a debate that sub-lobar resection isn’t significant difference to lobectomy. This large population-based study evaluated LCSS between a balanced cohort of 3,504 stage IA NSCLC patients with a tumor size less than or equal to 3 cm who underwent lobectomy and sub-lobar resection. According to the staging guidelines of the IASLC that T1 (≤ 3 cm) tumors should be further classified into three subgroups with 1-cm cut-off points (15), great interest for elderly individuals has been raised about whether there is any difference in the choice of surgical approach for NSCLC tumors ≤ 1 cm, > 1 to 2 cm and > 2 to 3 cm. The elderly patients usually have a declined cardiopulmonary reserve and limited life expectancy, surgical procedure with limited resection is supported by reducing morbidity and preserving limited lung function preservation. Since the LCSS is the most appropriate endpoint to determine the oncologic efficacy, we considered LCSS as the study indicator to compare the difference of surgical approaches for elder patients. This study revealed that sub-lobar resection did not provide a significant difference in LCSS to lobectomy in elderly patients with stage IA NSCLC if lymph node assessment is performed adequately. Similarly, there was no significant difference in LCSS between segmentectomy and wedge resection.

Some studies have attempted to evaluate the survival difference between lobectomy and sub-lobar resection for the patients with early stage NSCLC (7, 10, 17–19). The studies of Cao et al. and Dai et al. included the patients of all ages, while our study focused only the elderly patients (70 years and older). In addition, we performed PSM analysis to balance the baseline characteristics to lower the selection bias, however Dai et al. did not. Razi et al. found that sub-lobar resection is not inferior to lobectomy for T1a N0 M0 NSCLC in the elderly (20). Their study chose the patients with aged ≥ 75 y and didn’t use PSM. In contrast, we chose the old patients ≥70 years and the number of patients is much higher than Razi study. However, our study used PSM to balance the pretreatment variables to lower the selection bias.

Limited resection has been reported to preserve lung function without detriments to survival compared with lobectomy in stage I NSCLC tumors of 2 cm or less (21). Similarly, a study found similar LCSS between lobectomy and segmentectomy in a subset of the elderly patients (75 years and older) (22). However, we chose the old patients ≥70 years and the number of patients is much higher than Moon and colleagues’ study. More importantly, the examination of lymph nodes involvement is an important prognostic factor in early-stage NSCLC. We also explored the impact of lymph nodes on patient survival in our study, however the previous study did not consider this factor. The limited resection of the pulmonary parenchyma has advantages in terms of postoperative lung function (23). Zhang and colleagues found better LCSS with lobectomy *vs* segmentectomy after propensity matching in patients with <3 cm tumors (24). The data they choose included radiation. On the contrary, Patients we choose were excluded if they received chemotherapy or radiotherapy preoperatively, intraoperatively, or postoperatively. We only focus on the effect of surgical procedures on pathological outcomes. Similarly, a study found better LCSS with lobectomy in in elderly patients after propensity matching (25), the size of tumor they chose was less than or equal to 5 cm. Instead, the patients we chose were the stage IA NSCLC according to the latest eight edition of IASLC lung cancer staging project. The Alliance/CALGB 1405032 study also showed that perioperative mortality and morbidity did not seem to differ between lobar and sub-lobar resection for patients with NSCLC tumors ≤ 2 cm (9). A retrospective study showed that limited resection for small-sized lung cancer with ground-glass opacity (GGOs) was safe without any recurrence, and postoperative pulmonary function was well preserved (26). Research has demonstrated that patients with GGO-dominant clinical stage IA (≤ 2 cm) adenocarcinomas can be successfully treated with sub-lobar resection (27). Recently, a meta-analysis showed that no statistically significant difference was found for elderly patients with stage I NSCLC between sub-lobar resection and lobectomy in terms of the 1-, 3-, and 5-year survival rates (28). In addition, sub-lobar resection can improve patients’ postoperative quality of life by preserving pulmonary function and reducing the rates of morbidity and disability (13). However, the importance of lymph node (LN) dissection for early-stage NSCLC needs to be established. Several previous studies also showed that ≥ 4 regional LNs have with better survival rates in patients who undergo sub-lobar resection for stage IA NSCLC (29). An increased number of dissected LNs were associated with a lower risk of undiscovered positive lymph nodes, which may lead to a more accurate staging and a better survival (30, 31). However, we did not observe a significant difference in terms of LCSS for patients with no-LN dissection or ≥ 4 regional LNs in our study. This may result from the limited patient number in the group of no-LN dissection in the SEER database. We still strongly recommended a thorough regional LNs dissection based on the current knowledge.

In our study, which was based on a large population, we showed that there was no difference in LCSS between sub-lobar resection and lobectomy in elderly patients with stage IA NSCLC after PSM if lymph node assessment is performed adequately. In terms of histology, there was no statistical difference for ADC and OC patients in LCSS. For SQC patients, although lobectomy was associated with an LCSS superior to sub-lobar resection in the SQC, no significant statistical difference was found by adjusted subgroup analysis. In terms of subgroup analysis of tumor size (≤ 1 cm, > 1 to 2 cm and > 2 to 3 cm), our study revealed that there were no significant differences in the LCSS for NSCLC with tumor size ≤ 1 cm, > 1 to 2 cm and > 2 to 3cm. In addition, the multivariate analyses, which were used to reduce bias for our retrospective study, showed that older age, male gender, poor/undifferentiated grade, SQC histology type and larger tumor size were the independent prognostic factors, which could be predicted a worse LCSS.

It is reasonable to assume that local excision could be adequate for early stage lung cancer patients, especially elderly patients. As a consequence of population ageing, the number of elderly lung cancer patients (aged >70 years) is increasing rapidly worldwide, but evidence to guide appropriate treatment decisions for this group is generally limited (1). For elderly early-stage NSCLC patients, especially those who have poor pulmonary function or abnormal cardiac history, it is logical to exploit limited surgical management that preserves normal lung tissue, shortens the operation time and decreases postoperative complications (32). A multicenter retrospective studies demonstrated that short-term survival analysis showed no significant difference, and significant perioperative advantages were found in elderly patients with clinical stage I NSCLC who underwent sub-lobar resection (18). In this study, in terms of LCSS, it was well demonstrated that there was no obvious difference between lobectomy and sub-lobar resection for elderly patients with NSCLC tumors ≤ 3 cm as well as each subset (T ≤ 1 cm, > 1 to 2 cm and > 2 to 3 cm). In addition, sub-lobar resection was further classified into two subgroups, wedge resection and segmentectomy, which also showed no significant difference in LCSS. Similarly, subgroup analysis also demonstrated that no significant difference was found in histologic subtype or tumor sizes.

This study has several limitations of note. Firstly, based on the use of a retrospective study, some inherent biases (such as tumor location: central *vs*. peripheral) were inevitable, although adjustment by PSM was performed. Some features of the tumor, e.g., the solid or semi-solid (as well as the proportion of solid component size), could not be analyzed since the SEER database failed to provide this information. According to the eighth TNM classification system, the proportion of solid component size, rather than the whole tumor size, is considered a better measurement for T staging and prognostic predictions (33, 34). Secondly, the SEER database fails to provide the accurate information for peri-operative chemo- or radiotherapy. Moreover, the data of resection margins (R0, R1 or R2) are not provided in the SEER database. Last, information regarding surgical procedure (traditional open or minimally invasive approach), comorbidities and pulmonary function are not available in the SEER database. All of these issues could not be included in the PSM analysis, which need to be addressed in the further studies.

In conclusion, in terms of LCSS, lobectomy has no significant advantage over sub-lobar resection in elderly patients with stage IA NSCLC if lymph node assessment is performed adequately. The present data may contribute to develop a more suitable surgical treatment strategy for the stage IA elderly NSCLC patients.

## Data Availability Statement

Publicly available datasets were analyzed in this study. This data can be found here: Surveillance, Epidemiology, and End Results (SEER) database (https://seer.cancer.gov/).

## Author Contributions

BZ: Conceptualization; Data selection; Data analysis; Project administration; Writing—original draft; Writing—review and editing. RL: Conceptualization; Data analysis; Writing—original draft. DR: Conceptualization; Writing—original draft. XL: Data selection; Data analysis. YW: Data selection; Data analysis. HH: Data Analysis. SZ: Data Analysis. JC: Conceptualization; Supervision. ZS: Conceptualization; Supervision. SX: Conceptualization; Writing—review and editing; Supervision. All authors contributed to the article and approved the submitted version.

## Funding

The present study was funded by the National Natural Science Foundation of China (No. 81772464), the Tianjin Key Project of Natural Science Foundation (No.17JCZDJC36200), and Tianjin Science and Technology Plan Project (19ZXDBSY00060).

## Conflict of Interest

The authors declare that the research was conducted in the absence of any commercial or financial relationships that could be construed as a potential conflict of interest.

## Supplementary Material

Supplementary Figure 1 Subgroup analysis by independent review undergoing segmentectomy (Seg) and wedge resection (Wed).

Supplementary Figure 2 Lung cancer–specific survivals in patients with NSCLC ≤ 3 cm undergoing segmentectomy, or wedge resection before PSM **(A)** or after PSM **(B)**.

Supplementary Figure 3 Lung cancer–specific survivals in patients with NSCLC after PSM (NSCLC) ≤ 1 cm (A), NSCLC > 1 to 2 cm **(B)** or NSCLC > 2 to 3 cm **(C)** undergoing segmentectomy, or wedge resection.

Supplementary Figure 4 Lung cancer–specific survivals in patients with NSCLC after PSM [Histology type: SQC **(A)**, ADC **(B)** or OC **(C)**] undergoing segmentectomy, or wedge resection.

Supplementary Figure 5 Subgroup analysis of cancer-specific survival after PSM following segmentectomy and wedge resection for lung cancer with tumor size ≤ 3 cm. [Lymph nodes (LN) resection: no LN resection **(A)**, 1-3 LN resection **(B)** or ≥ 4 LN resection **(C)**].

Supplementary Figure 6 Univariate **(A)** and multivariate **(B)** Cox regression analysis of factors affecting lung cancer-specific survivals.

## Abbreviations

HR, hazard ratio; LCSS, lung cancer–specific survival; NSCLC, non–small cell lung cancer; SEER, Surveillance, Epidemiology, and End Results; PSM, propensity score matching; CI, confidence interval; SQC, squamous cell carcinoma; ADC, adenocarcinoma; OC other carcinoma.
