# Supplementary figures and images for: Comparison of Lobectomy and Sublobar Resection for Stage IA Elderly NSCLC Patients (≥70 Years): A Population-Based Propensity Score Matching’s Study
Source: Front Oncol. 2021 May 7;11:610638. doi: 10.3389/fonc.2021.610638 (PMC8139614; doi:10.3389/fonc.2021.610638)

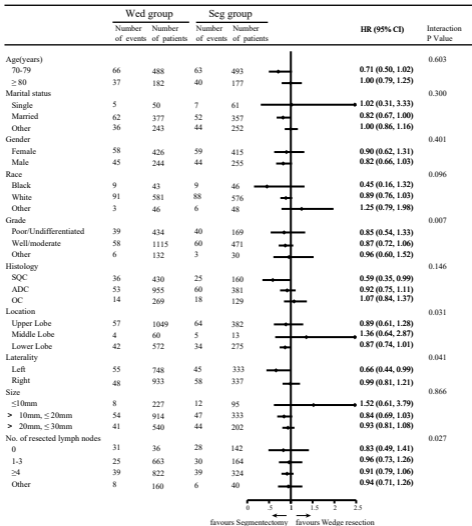

Supplement: Supplementary Figure 1 — Subgroup analysis by independent review undergoing segmentectomy (Seg) and wedge resection (Wed). [file Image_1.pdf]

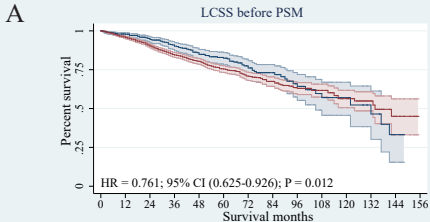

Number at risk

|                 |      |      |      |      |     |     |     |     |     |    |    |    |   |   |
|-----------------|------|------|------|------|-----|-----|-----|-----|-----|----|----|----|---|---|
| Segmentectomy   | 671  | 615  | 471  | 349  | 245 | 180 | 116 | 61  | 39  | 23 | 13 | 9  | 3 | 0 |
| Wedge resection | 2541 | 2302 | 1802 | 1341 | 972 | 625 | 368 | 192 | 128 | 88 | 45 | 22 | 8 | 0 |

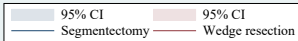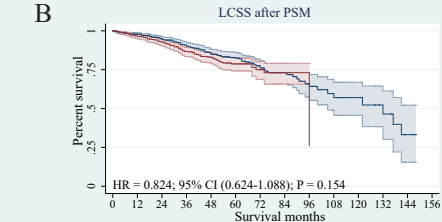

Number at risk

|                 |     |     |     |     |     |     |     |    |    |    |    |   |   |   |
|-----------------|-----|-----|-----|-----|-----|-----|-----|----|----|----|----|---|---|---|
| Segmentectomy   | 670 | 614 | 471 | 349 | 245 | 180 | 116 | 61 | 39 | 23 | 13 | 9 | 3 | 0 |
| Wedge resection | 670 | 616 | 488 | 345 | 233 | 123 | 45  | 3  | 1  | 0  | 0  | 0 | 0 | 0 |

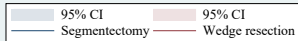

Supplement: Supplementary Figure 2 — Lung cancer–specific survivals in patients with NSCLC ≤ 3 cm undergoing segmentectomy, or wedge resection before PSM (A) or after PSM (B). [file Image_2.pdf]

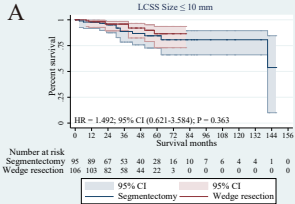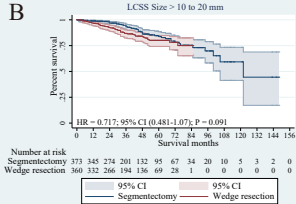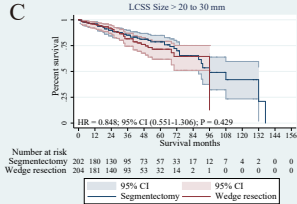

Supplement: Supplementary Figure 3 — Lung cancer–specific survivals in patients with NSCLC after PSM (NSCLC) ≤ 1 cm (A), NSCLC > 1 to 2 cm (B) or NSCLC > 2 to 3 cm (C) undergoing segmentectomy, or wedge resection. [file Image_3.pdf]

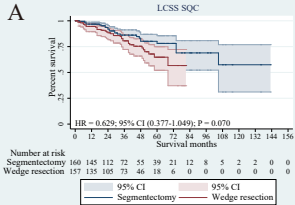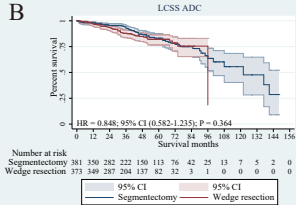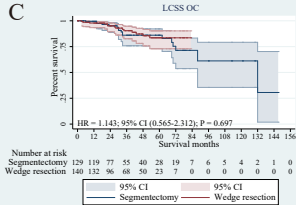

Supplement: Supplementary Figure 4 — Lung cancer–specific survivals in patients with NSCLC after PSM [Histology type: SQC (A), ADC (B) or OC (C)] undergoing segmentectomy, or wedge resection. [file Image_4.pdf]

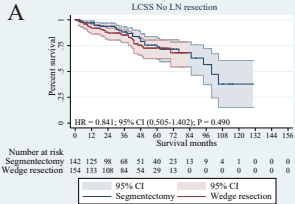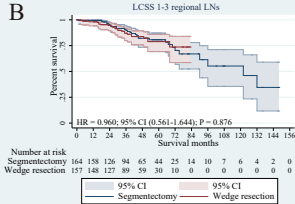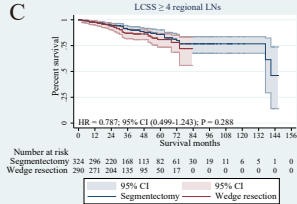

Supplement: Supplementary Figure 5 — Subgroup analysis of cancer-specific survival after PSM following segmentectomy and wedge resection for lung cancer with tumor size ≤ 3 cm. [Lymph nodes (LN) resection: no LN resection (A), 1-3 LN resection (B) or ≥ 4 LN resection (C)]. [file Image_5.pdf]
