# Supplementary material for: Comparison of Lobectomy and Sublobar Resection for Stage IA Elderly NSCLC Patients (≥70 Years): A Population-Based Propensity Score Matching’s Study
Source: Front Oncol. 2021 May 7;11:610638. doi: 10.3389/fonc.2021.610638 (PMC8139614; doi:10.3389/fonc.2021.610638)

A

Univariate COX Regression Analysis

Variable	LCSS	
	HR (95% CI)	p
Age (80+y vs ≥ 70 to 79 y)	**1.97 (1.48, 2.61)**	< 0.001
Marital status		0.068
Marital vs Signal	**1.22 (0.67, 2.21)**	0.512
Other vs Signal	**1.30 (0.71, 2.39)**	0.394
Gender(Male vs female)	**1.38 (1.05, 1.82)**	0.022
Race		0.106
White vs Black	**0.69 (0.42, 1.12)**	0.129
Other vs Black	**0.43 (0.19, 0.96)**	0.038
Grade		< 0.001
Well/moderate vs Poor/Undifferentiated	**0.45 (0.34, 0.60)**	< 0.001
Other vs Poor/Undifferentiated	**0.42 (0.21, 0.84)**	0.014
Location		0.487
Middle lobe vs Upper lobe	**1.14 (0.58, 2.24)**	0.712
Lower lobe vs Upper lobe	**0.86 (0.64, 1.14)**	0.286
Laterallity(Right vs Left)	**1.00 (0.76, 1.31)**	0.976
Histologic Type		0.015
ADC vs SQC	**0.65 (0.48, 0.89)**	0.008
OCvs SQC	**0.61 (0.40, 0.94)**	0.024
Size		< 0.001
> 1 to 2 cm vs ≤ 1cm	**1.49 (0.92, 2.42)**	0.104
> 2 to 3 cm vs ≤ 1cm	**2.44 (1.50, 3.98)**	< 0.001
Surgery type(L vs Sub-L)	**0.81 (0.61, 1.07)**	0.140
Scope Reg LN Sur		0.047
1-3 vs Non	**0.77 (0.54, 1.12)**	0.175
> 4 vs Non	**0.62 (0.44, 0.87)**	0.006
Other vs Non	**0.64 (0.36, 1.14)**	0.131

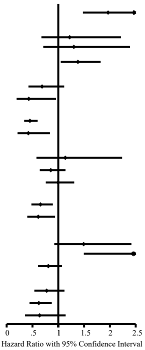

B

Multivariate COX Regression Analysis

Variable	LCSS	
	HR (95% CI)	p
Age (≥ 80+ vs ≥ 70 to 79y y)	**1.78 (1.34, 2.38)**	< 0.001
Gender(Male vs Female)	**1.22 (0.93, 1.62)**	0.157
Grade		< 0.001
Well/moderate vs Poor/Undifferentiated	**0.51 (0.37, 0.69)**	< 0.001
Other vs Poor/Undifferentiated	**0.53 (0.26, 1.08)**	0.080
Histologic Type		0.175
ADC vs SQC	**0.85 (0.61, 1.17)**	0.306
OC vs SQC	**0.66 (0.43, 1.02)**	0.064
Size		0.003
> 1 to 2 cm vs ≤ 1cm	**1.30 (0.80, 2.11)**	0.298
> 2 to 3 cm vs ≤ 1cm	**1.99 (1.21, 3.28)**	0.007
Scope Reg LN Sur		0.130
1-3 vs Non	**0.81 (0.56, 1.17)**	0.264
>4 vs Non	**0.67 (0.47, 0.94)**	0.022
Other vs Non	**0.68 (0.38, 1.21)**	0.190

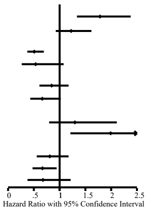

Supplement: Supplementary Figure 6 — Univariate (A) and multivariate (B) Cox regression analysis of factors affecting lung cancer-specific survivals. [file Image_6.pdf]
