# Supplementary material for: Comparison of Lobectomy and Sublobar Resection for Stage IA Elderly NSCLC Patients (≥70 Years): A Population-Based Propensity Score Matching’s Study
Source: Front Oncol. 2021 May 7;11:610638. doi: 10.3389/fonc.2021.610638 (PMC8139614; doi:10.3389/fonc.2021.610638)
Supplement: Supplementary file 7 [file Table_1.docx]

| Supplementary Table 1 Baseline Characteristics for Patients With NSCLC ≤ 3 cm | | | | | | | | |  |
| --- | --- | --- | --- | --- | --- | --- | --- | --- | --- |
|  | No. (%) of Patients before PSM | | | | No. (%) of Patients after PSM | | | | |
|  | Wed(N=2541) | Seg(N=671) | P | Smd | Wed(N=670) | Seg(N=670) | P | Smd | |
| Age (Median) |  |  | 0.155 |  |  |  | 0.758 |  | |
| ≥ 70 to 79y | 1796(70.6) | 493(73.5) |  | -0.064 | 488(72.9) | 493(73.5) |  | -0.014 | |
| ≥ 80y | 745(29.4) | 178(26.5) |  | 0.064 | 182(27.1) | 177(26.5) |  | 0.014 | |
| Marital status |  |  | 0.018 |  |  |  | 0.407 |  | |
| Single | 160(6.3) | 62(9.2) |  | -0.108 | 50(7.5) | 61(9.1) |  | -0.058 | |
| Married | 1345(52.9) | 357(53.3) |  | -0.008 | 377(56.2) | 357(53.3) |  | 0.058 | |
| Other | 1036(40.8) | 252(37.5) |  | 0.067 | 243(36.3) | 252(37.6) |  | -0.026 | |
| Gender |  |  | 0.143 |  |  |  | 0.534 |  | |
| Female | 1496(58.8) | 416(61.9) |  | -0.063 | 426(63.5) | 415(62) |  | 0.031 | |
| Male | 1045(41.2) | 255(38.1) |  | 0.063 | 244(36.5) | 255(38) |  | -0.031 | |
| Race |  |  | 0.518 |  |  |  | 0.921 |  | |
| Black | 146(5.8) | 46(6.8) |  | -0.041 | 43(6.5) | 46(6.8) |  | -0.049 | |
| White | 2222(87.4) | 577(86) |  | 0.041 | 581(86.7) | 576(86.1) |  | 0.017 | |
| Other | 173(6.8) | 48(7.2) |  | -0.015 | 46(6.8) | 48(7.1) |  | -0.011 | |
| Grade |  |  | 0.001 |  |  |  | 0.025 |  | |
| Poor/Undifferentiated | 626(24.6) | 170(25.4) |  | -0.018 | 165(24.7) | 169(25.2) |  | -0.011 | |
| Well/moderate | 1690(66.6) | 471(70.2) |  | -0.077 | 451(67.3) | 471(70.2) |  | -0.062 | |
| Other | 225(8.8) | 30(4.4) |  | 0.177 | 54(8) | 30(4.6) |  | 0.140 | |
| Histology |  |  | 0.188 |  |  |  | 0.755 |  | |
| SQC | 684(26.9) | 160(23.9) |  | 0.068 | 157(23.4) | 160(23.8) |  | -0.009 | |
| ADC | 1422(55.9) | 382(56.9) |  | -0.020 | 373(55.8) | 381(56.8) |  | -0.020 | |
| OC | 435(17.2) | 129(19.2) |  | -0.051 | 140(20.8) | 129(19.4) |  | 0.034 | |
| Location |  |  | < 0.001 |  |  |  | 0.003 |  | |
| Upper Lobe | 1576(62) | 383(57.1) |  | 0.099 | 378(56.6) | 382(57) |  | -0.008 | |
| Middle Lobe | 128(5.1) | 13(2) |  | 0.168 | 36(5.1) | 13(2) |  | 0.168 | |
| Lower Lobe | 837(32.9) | 275(40.9) |  | -0.166 | 256(38.3) | 275(41) |  | -0.055 | |
| Laterality |  |  | 0.005 |  |  |  | 0.548 |  | |
| Left | 1109(43.7) | 334(49.7) |  | -0.120 | 322(48.1) | 333(49.7) |  | -0.032 | |
| Right | 1432(56.3) | 337(50.3) |  | 0.120 | 348(51.9) | 337(50.3) |  | 0.032 | |
| Size |  |  | 0.001 |  |  |  | 0.656 |  | |
| ≤10mm | 490(19.3) | 95(14.2) |  | 0.136 | 106(15.8) | 95(14.3) |  | 0.041 | |
| ＞10mm, ≤20mm | 1420(55.9) | 373(55.6) |  | 0.006 | 360(53.8) | 373(55.6) |  | -0.036 | |
| ＞20mm, ≤30mm | 631(24.8) | 203(30.2) |  | -0.121 | 204(30.4) | 202(30.1) |  | 0.006 | |
| No. of resected lymph nodes |  |  | < 0.001 |  |  |  | 0.017 |  | |
| 0 | 1191(46.9) | 142(21.3) |  | 0.560 | 154(22.9) | 142(21.1) |  | 0.043 | |
| 1-3 | 607(23.8) | 164(24.4) |  | -0.014 | 157(23.4) | 164(24.4) |  | -0.023 | |
| ≥4 | 627(24.7) | 324(48.2) |  | -0.503 | 290(43.5) | 324(48.3) |  | -0.096 | |
| Other | 116(4.6) | 41(6.1) |  | -0.066 | 69(10.2) | 40(6.2) |  | 0.146 | |

Seg: Segmentectomy, Wed: wedge resection, SQC: Squamous carcinoma, ADC: adenocarcinoma, OC: other carcinoma, PSM: propensity score matching, Smd: standardized mean differences
